# Improved Biobutanol Production in 2-L Simultaneous Saccharification and Fermentation with Delayed Yeast Extract Feeding and *in-situ* Recovery

**DOI:** 10.1038/s41598-019-43718-1

**Published:** 2019-05-15

**Authors:** Muhammad Siddiq Mohamed Salleh, Mohamad Faizal Ibrahim, Ahmad Muhaimin Roslan, Suraini Abd-Aziz

**Affiliations:** 1 Department of Bioprocess Technology, Faculty of Biotechnology and Biomolecular Sciences, Universiti Putra Malaysia, 43400 UPM Serdang, Selangor, Malaysia; 2 Laboratory of Biopolymer and Derivatives, Institute of Tropical and Forestry and Forest Product, 43400 UPM Serdang, Selangor, Malaysia

**Keywords:** Environmental biotechnology, Applied microbiology

## Abstract

Simultaneous saccharification and fermentation (SSF) with delayed yeast extract feeding (DYEF) was conducted in a 2-L bioreactor equipped with *in-situ* recovery using a gas stripping in order to enhance biobutanol production from lignocellulosic biomass of oil palm empty fruit bunch (OPEFB). This study showed that 2.88 g/L of biobutanol has been produced from SSF with a similar yield of 0.23 g/g as compared to separate hydrolysis and fermentation (SHF). An increase of 42% of biobutanol concentration was observed when DYEF was introduced in the SSF at 39 h of fermentation operation. Biobutanol production was further enhanced up to 11% with a total improvement of 72% when *in-situ* recovery using a gas stripping was implemented to reduce the solvents inhibition in the bioreactor. In overall, DYEF and *in-situ* recovery were able to enhance biobutanol production in SSF.

## Introduction

Biobutanol is one of promising alternative biofuel as the demand for renewable and alternative energy increases in recent years. Biobutanol makes it’s appealing to researchers as it has higher energy content and lowers volatility as compared to bioethanol and biomethanol. Besides, biobutanol also has similar characteristics with gasoline, thus, it can be used in car engines and distribution system without any modifications^1^. This C_4_ alcoholic compound is a colourless liquid with a distinct odour and it is completely miscible with organic solvents and partly miscible with water^2–4^. Currently, butanol is produced through a petrochemical process and it is used as chemical feedstock in the plastic industry, paints, coatings, plasticizers, adhesives and food extractant^5^. The global market demand for butanol is pegged at 2.8 metric tonnes per year, with a market value of USD 4.2 billion. The International Energy Agency forecasts biofuels demand to increase from 60 million tonnes per year in 2008 to 690 million tonnes per year in 2050 with the market value increases by 3.2% in 2025^6^.

In order to meet the energy demand, biobutanol produced from renewable resources such as lignocellulosic biomass is now under-focused. In Malaysia, oil palm empty fruit bunch (OPEFB) is one of the most abundant lignocellulosic biomass being produced with 6.61 million tonnes per year^7^. This value is estimated to increase up to 100 million tonnes in 2020^8^. OPEFB contains 39% of cellulose, 21% of hemicellulose and 19% of lignin^9^. It could be reached up to 84% of total potential sugars (cellulose + hemicellulose) after pretreatment^1^. However, utilising OPEFB as raw material for biobutanol production has several challenges including multiple processing steps, low biobutanol concentration and yield^1,10–12^. In order to overcome these problems, several bioprocessing strategies were performed and evaluated in this study.

Simultaneous saccharification and fermentation (SSF) process can be applied to combine the process of saccharification and fermentation in a single reactor at the same processing time, thus, reducing the cost of materials, apparatus, time and labour, and subsequently improve the whole process productivity from saccharification to fermentation. In addition, biobutanol production (concentration and yield) through SSF is also comparable to separate hydrolysis and fermentation (SHF) as reported by Ibrahim *et al*.^13^. However, one of the major problems in acetone-ethanol-butanol (ABE) fermentation is the production of acids (acetic and butyric), which is double to the production of biobutanol^14^. It was reported that manipulation on feeding time of yeast extract during fermentation able to shorten the transition phase between acidogenesis to solventogenesis and enhance the acids re-assimilation for butanol production^15^. Therefore, delayed yeast extract feeding (DYEF) was introduced in the SSF process as it could enhance the biobutanol concentration and yield. The process was further enhanced by integrating the *in-situ* recovery using a gas stripping to reduce solvent inhibition that limits biobutanol formation by the cells.

## Materials and Method

### Substrate preparation and pretreatment

Pressed and shredded oil palm empty fruit bunch (OPEFB) was obtained from Dengkil Palm Oil Mill, Hulu Langat, Selangor, Malaysia. The OPEFB was ground to a size of 3–15 mm using a hammer mill. Then, the OPEFB was soaked overnight and washed with commercial detergent to remove dust and oil. The washed OPEFB was dried in an oven at 60 °C for 24 h. A 100 g of washed and dried OPEFB was soaked in 2 L of 2% NaOH for 4 h followed by autoclaving at 121 °C, for 5 min before it was washed again with tap water to remove alkaline residue until almost neutral pH was obtained^16^. The pretreated OPEFB was dried in an oven at 60 °C for 24 h and stored in a sealed plastic container prior to the saccharification and SSF.

### Enzymatic saccharification

Commercial cellulase (Celluclast 1.5 L, Novozymes, Denmark) was used for the saccharification of pretreated OPEFB into fermentable sugar. The saccharification was conducted at 1.5 L working volume in a 2-L bioreactor (EYELA, Japan) with an agitation speed of 150 rpm, the temperature at 35 °C for 96 h. The process was conducted by adding 5% of pretreated OPEFB into the 2-L bioreactor and autoclaved at 121 °C for 15 min. Approximately 1.5 L of sterilized 0.05 M acetate buffer, pH 5.5 was added into the bioreactor. Then, 15 FPU/mL of prepared cellulase solution sterilized via filtration using 0.22 mm sterilized nylon driven filters (Millipore, Denmark) was added into the bioreactor before sparged with nitrogen gas for about 1 h until no oxygen was detected. Samples were collected every 24 h and kept in the freezer of −20 °C for sugar analysis.

### Inoculum preparation

The stock culture of *Clostridium acetobutylicum* ATCC 824 purchased from American Type Culture Collection (ATCC) was anaerobically transferred into 100 mL of Reinforced Clostridial Medium (Merck, Germany). The cultured medium was incubated in Memmert incubator at 37 °C for 24 h under static condition.

### Fermentation

#### Separate hydrolysis and fermentation

SHF was conducted using fermentable sugar obtained from the hydrolysis of pretreated OPEFB. The sugar was autoclaved at 110 °C for 5 min. Then, 6 g/L of pre-sterilized yeast extract and 2 mL of each P2-medium components^13^ were added into the solution and sparged with nitrogen gas for about 1 h until no oxygen was detected. The fermentation was initiated by inoculating 15% of prepared inoculum (OD_620_ set at 1.0) into the bioreactor^17^. The inoculated media were incubated at 35 °C with an agitation speed of 150 rpm for 120 h. Two-ml of the liquid sample was withdrawn from the bioreactor and kept at −20 °C prior to sample analysis.

#### Simultaneous saccharification and fermentation

This SSF process was conducted based on the optimal conditions reported by Razali *et al*.^17^. SSF was conducted by adding 5% of pretreated OPEFB into a 2-L bioreactor and autoclaved at 121 °C for 15 min. A 6 g/L of sterilized yeast extract solution and 0.05 M of acetate buffer pH 5.5 were added into the bioreactor. Then, 15 FPU/mL of prepared cellulase was sterilized via filtration using 0.22 mm sterilized nylon driven filters (Millipore, Denmark) before it was added into a bioreactor together with 2 mL of each P2 medium component. All the mixtures were sparged with nitrogen gas for about 1 h until no oxygen was detected. The SSF was initiated by inoculating 15% of prepared inoculum (with the OD_620_ set at 1.0) into the bioreactor. The inoculated medium was incubated at 35 °C with an agitation speed of 150 rpm for 144 h. A 2 ml of the liquid sample from each fermentation bottle was withdrawn using a syringe and kept at −20 °C prior to sample analysis.

#### Simultaneous saccharification and fermentation with delayed yeast extract feeding

The SSF with DYEF was conducted similarly to the SSF process except the yeast extract was fed after 39 h of fermentation was operated^15^.

#### *In-situ* recovery using gas stripping

The *in-situ* recovery using a gas stripping technique was conducted intermittently after 48 h of fermentation duration following the procedures by Xue *et al*.^18^. A set of condenser unit (Pyrex Graham coil, 30 × 300 mm) purchased from Fisher Scientific was autoclaved separately for 15 min at 121 °C, and then aseptically connected to the bioreactor unit, pump and recovery tank. Then, the whole system was sparged with nitrogen gas to ensure the oxygen-free environment before the fermentation was started. After 48 h of fermentation, the gas stripping technique was carried out by circulating the nitrogen gas (off-gas) at 1.5 L/min that passed through the fermentation broth. The peristaltic pump connected to the condenser was turned on when the butanol concentration inside the fermentation broth increased to ~1 g/L, which is after 48 h of fermentation. The condenser temperature was maintained at 2 °C to allow butanol to condense and collected in a flask that immersed with cold water. This stripping process was conducted for 6 h and was continued again on the next day until the end of the fermentation.

## Results and Discussion

### Enzymatic saccharification of pretreated OPEFB

**Figure 1:**
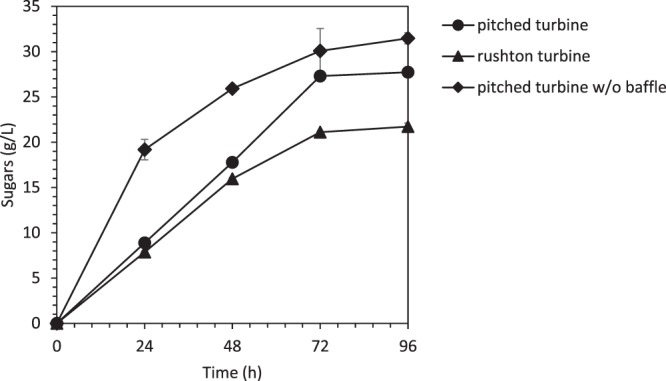
Effect of different impellers, with and without baffle on the enzymatic saccharification of pretreated oil palm empty fruit bunch in a 2-L bioreactor.

The upscale of the saccharification and the fermentation processes from serum bottle to bioreactor required modification on the mixing process since a larger amount of biomass is used. In this study, the major challenges occurred since the SSF process is using the solid medium of OPEFB loaded in the bioreactor instead of sugar in a liquid form. In order to make sure the OPEFB is well homogenized in the bioreactor, a study on the saccharification efficiency in the bioreactor was conducted with the aim to get at least similar sugar concentration of 30 g/L as obtained from the saccharification in serum bottle that was conducted by Ibrahim *et al*.^13^. Two types of impeller were tested, i.e.: Rushton and pitched turbine, which produced 21.11 and 27.30 g/L of sugar, respectively as shown in Fig. 1. It should be noted that the optimal sugar concentration for ABE fermentation by Clostridia is at 60 g/L^1^. The sugar should not be less than 40 g/L or else the cells will be triggered to produce more acids instead of solvents^19^. However, OPEFB loading of more than 5% will reduce the mixing efficiency and cause the inability of the cells to withstand at high substrate loading^17^. Therefore, this present study utilized 5% of OPEFB in the whole process after being optimised by Razali *et al*.^17^.

Saccharification using Rushton turbine (common impeller for fermentation) produced the lowest sugar concentration due to improper mixing of OPEFB. Although the OPEFB size is in between 3–5 mm, the bulky and fibrous structure of OPEFB makes the mixing process inefficient. During the saccharification process, it was observed that the OPEFB was not homogeneously mixed as the OPEFB starts to accumulate at the bottom of the bioreactor as shown in supplementary document (S1). This observation was supported with the low sugar concentration obtained after 72 h of saccharification time, which was 21 g/L. Rushton turbine is a flat disk turbine that emits the radial flow^20^, which forced the OPEFB toward the wall of the bioreactor. Meanwhile, the gravitational force holds the OPEFB at the bottom of the bioreactor, which caused the OPEFB to accumulate. The accumulation of OPEFB at the bottom of the bioreactor hindered the accessibility of cellulase to act on OPEFB, which results in low sugar concentration produced in the system.

Therefore, the impeller was changed to the pitched turbine, with 45° angle to pull the substrate upward and circulate within the bioreactor. It was observed that the axial flow performed by pitched turbine has a better mixing property as compared to radial flow by Rushton turbine. The sugar produced improved from 21 g/L (Rushton turbine) to 27 g/L (pitched turbine). However, there is some amount of OPEFB trapped at the baffle, at the bottom of the bioreactor. Therefore, the baffle has been removed, which had improved the mixing process. All OPEFB fibres were homogeneously mixed in the bioreactor and the sugar production has been increased to 31 g/L, which was similar to the saccharification conducted in a serum bottle and shake flask obtained by Ibrahim *et al*.^13^. The average saccharification rate for the first 24 h is 0.75 g/L.h and for 48 h is about 0.63 g/L.h. It should be noted that the saccharification is a rate-limiting step for the SSF. The pitched turbine which was enhanced the saccharification efficiency could provide enough sugar for the cells during SSF. Enough sugar supplied is important in ABE fermentation to prevent high acid production, which leads to an acid crash^21^.

### ABE fermentation

#### Separate hydrolysis and fermentation

**Figure 2:**
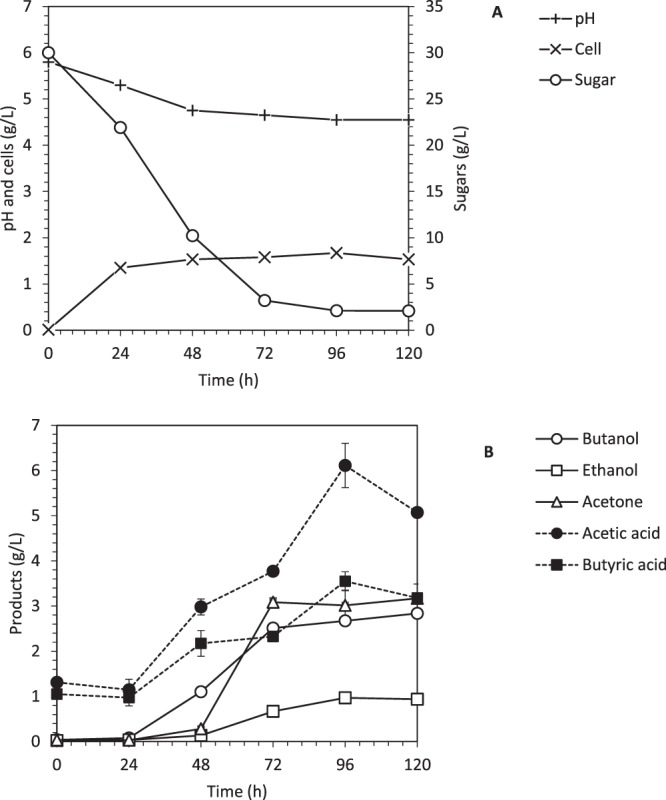
Separate hydrolysis and fermentation of oil palm empty fruit bunch hydrolysate in a 2-L bioreactor. (**A**) Profile of cells growth, glucose consumption, and pH and (**B**) Profile of biobutanol, ethanol, acetone, acetic and butyric acid production.

SHF was conducted using sugar obtained from the saccharification of pretreated OPEFB by cellulase as shown in Fig. 2. The main aim of conducting SHF in a 2-L bioreactor is to compare with SHF conducted in a 125-mL serum bottle^13,17^, as well as a control to be compared with the SSF process. In this study, approximately 30 g/L of the sugar from OPEFB was supplied in this fermentation because this is the highest sugar concentration that can be obtained from the saccharification of OPEFB, which was also reported by Ibrahim *et al*.^13^. After 72 h of fermentation duration, 2.51 g/L of biobutanol was produced with the yield of 0.10 g/g. It was also observed that 6.1 g/L of acetic acid and 3.5 g/L of butyric acid were produced in the system. High acid production caused the cells to experience the acid crash phenomenon that ceased the cell metabolism to continuously grow and produce the solvents^22^. The acid inhibition phenomenon was detected as there is remaining sugar (3.22 g/L) detected in the system even after 72 h of fermentation.

#### Simultaneous saccharification and fermentation

**Figure 3:**
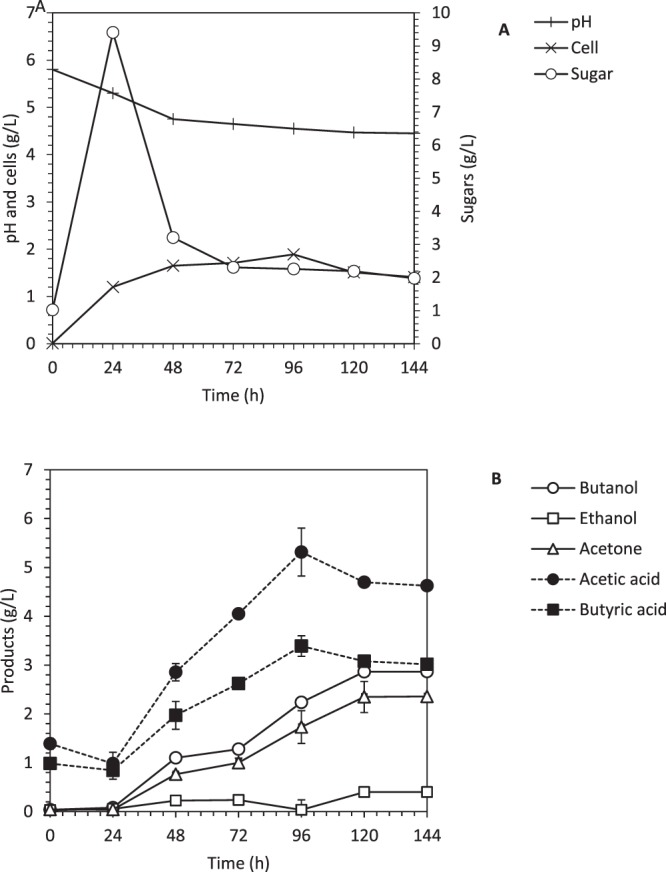
Simultaneous saccharification and fermentation of oil palm empty fruit bunch in a 2-L bioreactor. (**A**) Profile of cells growth, glucose consumption, and pH and (**B**) Profile of biobutanol production, ethanol production, acetone production, acetic acid production and butyric acid production.

SSF was conducted in a 2-L bioreactor using 5% pretreated OPEFB as a substrate. The SSF combines both saccharification and fermentation processes in a single operation at the same time and in a single vessel. Therefore, SSF reduces the number of steps, the whole operation duration, equipment and apparatus as compared to SHF^1^. The whole operation took 10 days for SHF including substrate preparation, saccharification and sugar recovery, medium and inoculum preparation, and ABE fermentation. The SSF reduces the operation duration to 6 days by operating the saccharification and fermentation process simultaneously. In this study, the SSF produced 2.88 g/L of biobutanol (Fig. 3), which is almost similar to the biobutanol production in SHF. This SSF operated in a bioreactor also produced a similar fermentation profile as compared to the SSF operated in serum bottles^13^. It should be noted that the SSF is successfully operated in the bioreactor after the improvement of the mixing process as discussed in Section 3.1.

The major challenge in this SSF is the low sugar concentration generated from 5% of OPEFB, which is at ~30 g/L. The substrate loading cannot be increased over 5% due to the bulky structure of OPEFB that occupies space and hinders the mixing process, resulting in low sugar production and yield^23^. Sugar concentration of lower than 40 g/L could cause the cells to produce more acids than solvents^19^. The cells actively consume the sugars for cell duplication, acids formation and hydrogen gas production during the log phase, usually within 48 h of fermentation time for SHF and 72 h for SSF. Since the low concentration of sugars was provided in the system, the remaining sugar after 72 h of fermentation is too low to support the solventogenic phase to produce solvents, resulted with a low amount of biobutanol detected in the fermentation broth and a longer transition state from acidogenesis to solventogenesis. This is because the metabolic activity of the cells required a suitable value of undissociated acids for transition phase of acidogenesis to solventogenesis, while at higher concentrations of acids (refers to acids in the protonated form) can cause the acid inhibition or “acid crash” phenomenon^22^. It was reported that the maximum acids threshold will be in the range of 5.0 to 5.3 g/L^22^. High accumulation of acids (7.61 g/L) can be observed in this study, which in this case the acids exceeds the threshold limit as the reason “acid crash” to occur. To overcome the acid inhibition in this SSF, the feeding time of yeast extract was delayed to 39 h as suggested by Li *et al*.^15^, who had tested to improve the transition from acidogenic to solventogenic phase in SHF using cassava as substrate. Similar conditions of solvent-producing cultures used for ABE production has shown the tendency to produce a high level of acids when dealing with the lignocellulosic substrate^24,25^.

#### Simultaneous saccharification and fermentation with delayed yeast extract feeding

**Figure 4:**
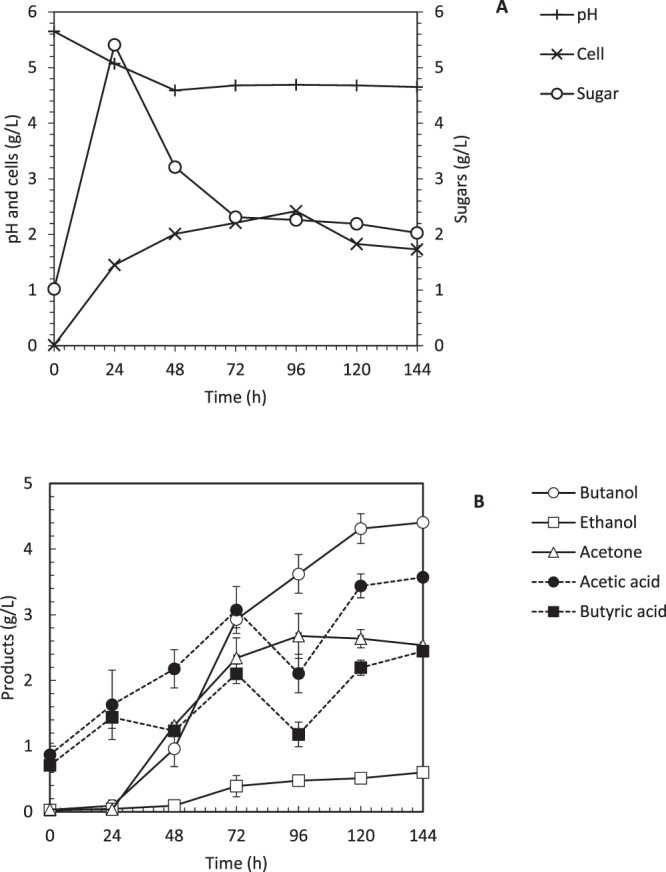
Simultaneous saccharification and fermentation (SSF) with delayed yeast extract feeding (DYEF) in the 2-L bioreactor. (**A**) Profile of cells growth, glucose consumption, and pH and (**B**) Profile of biobutanol, ethanol, acetone, acetic acid and butyric acid production.

The DYEF was introduced in ABE fermentation in order to improve the biobutanol production by improving the acids re-assimilation during the transition from acidogenesis to solventogenesis. Introducing DYEF at 39 h in SSF had increased the biobutanol production from 2.88 g/L to 4.31 g/L, which is equivalent to 45% increment. DYEF shortening the transition phase from acidogenesis to solventogenesis, thus prevent the acid crash to happen during the fermentation. This situation can be observed when the acid production (acetic and butyric) was kept hovering around 2 to 3 g/L, respectively as shown in Fig. 4, which is lower than the acids produced in normal SSF (above 4 g/L). The re-assimilation of acids can be clearly observed after 72 h of fermentation time, where the amount of acids was reduced, and the solvents production was increased. The pH profile showed a slight increment of +0.1, become pH 4.65 at 72 h until the end of fermentation. From 0 to 48 h, acids were produced, which indicates the acidogenic phase of the cells. The yeast extract was added at 39 h, which immediately shifted the phase to solventogenesis and increased the biobutanol production from 0.01 g/L at 24 h to 1.3 g/L at 48 h and soaring up to 3.2 g/L of biobutanol after 72 h of fermentation as shown in Fig. 4. The biobutanol yield (0.15 g/g) and productivity (0.043 g/L.h) is higher than normal SSF that has biobutanol yield and productivity of 0.12 g/g and 0.024 g/L.h, respectively. Addition of yeast extract at 39 h enhanced the expression of key enzymes that function to re-assimilate the acetic and butyric acid and shifted the phase to solvents production. CoA-transferase functions to re-assimilate the organic acid (acetic and butyric acid) and convert into acetyl-CoA and butyryl-CoA, respectively. The concentrations of histidine (histidine family), and threonine, lysine and methionine (aspartic acid family) in the broth increases after added with yeast extract (Li *et al*.^15^).

#### Simultaneous saccharification and fermentation with delayed yeast extract feeding and *in-situ* recovery

**Figure 5:**
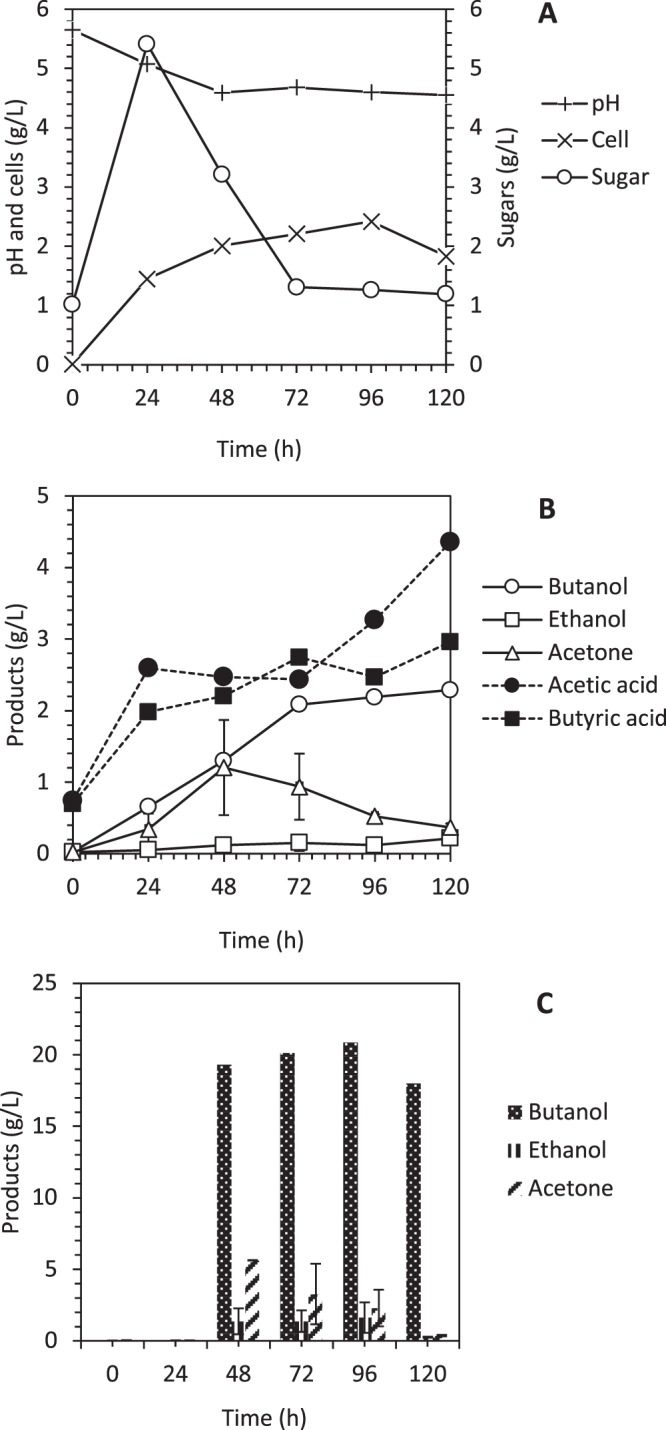
Simultaneous saccharification and fermentation with delayed yeast extract feeding and *in-situ* recovery using gas stripping. (**A**) Profile of cells growth, glucose consumption, and pH, (**B**) Profile of biobutanol production, ethanol production, acetone production, acetic acid production and butyric acid production and (**C**) acetone, butanol and ethanol recovery profile.

The SSF with DYEF was further improved by implementing *in-situ* recovery using a gas stripping technique to enhance biobutanol production. This technique serves as two-in-one benefits, which is for recovery and prevent the inhibitory product from further deteriorate the cell. Integrating *in-situ* recovery in SSF with DYEF had improved the biobutanol production by 11%, from 4.4 g/L to 4.96 g/L. Figure 5 shows that 2.21 g/L of biobutanol was produced after 48 h of fermentation. The amount of biobutanol inside the bioreactor was kept at 2.2–2.3 g/L after the *in-situ* recovery was operated at 48, 72, 96 and 120 h until the end of the fermentation. This experiment showed that the *in-situ* recovery is functional to be integrated with the SSF process and had improved the biobutanol production yield to 0.16 g/g as compared to 0.15 g/g operated in SSF with DYEF without *in-situ* recovery. The function of *in-situ* recovery to recover solvent from inhibiting the cells has been widely reported^26–30^. Therefore, integrating *in-situ* recovery in SSF with DYEF has successfully benefited the process to enhance the overall biobutanol production and yield.

In addition to the *in-situ* recovery process in SSF with DYEF, Fig. 5(C) shows the recovered products of acetone, butanol and ethanol. Results showed that the gas stripping technique capable to selectively recover biobutanol at a higher concentration as compared to acetone and ethanol. There is only a trace amount acid was detected in the recovery flask. Therefore, a high concentration of acids (4.5 g/L acetic acid, and 3.0 g/L butyric acid) was recorded in the fermentation vessel after 120 h of fermentation time. This is due to most of the biobutanol have been recovered and left acids in the vessel. It was 26 mL of recovered products were collected after 120 h of operation with the highest biobutanol concentration was recorded at 96 h (20.84 g/L). It was 83% purity of the recovered biobutanol with the remaining 17% are acetone and ethanol.

### Comparison of the processes

This study demonstrated the enhancement of biobutanol production by conducting SSF with DYEF and *in-situ* recovery. Process comparison in Table 1 shows that the DYEF has significantly improved the biobutanol production in both SHF and SSF processes by 44% and 53%, respectively, as compared to without DYEF. A high concentration of biobutanol in the fermentation broth was reduced when *in-situ* recovery was conducted using gas stripping. Integrating *in-situ* recovery in SHF and SSF with DYEF had enhanced overall biobutanol production to 4.60 g/L and 4.96 g/L, respectively. The DYEF with *in-situ* recovery also improved the biobutanol yield in SSF from 0.10 g/g to 0.16 g/g, which also significantly increased the biobutanol productivity from 0.29 to 0.56 g/L/h. This study demonstrated the capability of the cells to consume more sugar and transformed into butanol when the inhibitors (acids) are controlled using the DYEF technique and (solvents) are removed using *in-situ* recovery. The conditions provided to cells had improved the transition state from acidogenesis to solventogenesis that trigger the cells to produce more solvents. This study also proves the capability of conducting *in-situ* recovery in the SSF process whereby 26 mL of solvents containing ~20 g/L of biobutanol with 83% purity were recovered from the fermentation broth.

**Table 1:** Comparison of various ABE fermentation strategies by *Clostridium acetobutylicum* ATCC 824 using oil palm empty fruit bunch as a substrate.

Fermentation Strategies	SHF	SSF	SHF with DYEF	SSF with DYEF	SHF with DYEF and *in-situ* recovery	SSF with DYEF and *in-situ* recovery
Residual sugar, g/L	2.75	2.19	1.98	1.76	1.63	1.39
Fermentation time, h	120	120	120	120	120	120
Sugar consumption, g/L	27.25	29.82	28.02	29.71	28.37	30.08
Acetone, g/L	2.57	2.75	2.85	2.70	2.02	2.20
Biobutanol, g/L	2.86	2.88	4.13	4.41	4.60	4.96
Ethanol, g/L	0.10	0.29	0.35	0.50	1.50	0.99
Total ABE, g/L	5.74	6.55	7.33	7.41	8.12	8.15
Acetic acid, g/L	5.07	4.62	2.22	3.44	3.20	4.36
Butyric acid, g/L	3.18	3.01	2.09	2.19	2.56	2.96
Biobutanol yield, g/g	0.10	0.10	0.15	0.15	0.16	0.16
ABE yield, g/g	0.23	0.23	0.26	0.26	0.29	0.28
Biobutanol productivity, g/L/h	0.024	0.029	0.034	0.043	0.038	0.056
ABE productivity, g/L/h	0.047	0.068	0.061	0.077	0.067	0.085

In comparison with other studies (Table 2), the DYEF with *in-situ* recovery produced higher biobutanol yield as compared to other fermentation processes by *C*. *acetobutylicum* ATCC 824. It shows that the DYEF has high impact in controlling the transition state of acidogenesis into solventogenesis and trigger the cells to regulate the available sugar to produce more butanol, resulted in a high butanol yield of 0.16 g/g. The ABE fermentation using alkali-pretreated switch grass also yielded 0.16 g/g of butanol, with a higher butanol concentration of 7.8 g/L^31^. This study investigated the effect of Tween 80 in the fermentative butanol production, but using a higher substrate loading of 50 g/L. Other studies showed a lower butanol yield although a higher initial sugar was used as compared to this study. The variety of substrate characteristics and fermentation process might influence the cells to generate the products. Lignocellulosic materials such as OPEFB possess furan derivatives, aliphatic acids, phenolic and other aromatic compounds produced as by-products after the saccharification process^32^. These compounds can inhibit the cell performance and reduce it capability to consume more sugars and transform it into solvents. This factor might need to be considered when conducting the ABE fermentation using lignocellulosic material as substrate.

**Table 2:** Comparison of biobutanol production by *Clostridium acetobutylicum* ATCC 824 in various fermentation conditions.

Fermentation operations	Substrate conc.	Biobutanol			References
		Conc. (g/L)	Yield (g/g)	Productivity (g/L/h)	
SSF	30 g/L OPEFB	4.96	0.16	0.08	This study
SHF	30 g/L sago pith residue hydrolysate	2.23	0.10	0.03	Linggang *et al*.^33^
SHF	50 g/L sago pith residue hydrolysate	5.41	0.13	0.07	Linggang *et al*.^33^
SHF	60 g/L konjac waste	7.1	0.12	0.19	Shao & Chen,^34^
Glucose	70 g/L glucose	8.17	0.13	—	Razak *et al*.^35^
SHF	70 g/L OPDC hydrolysate	6.04	0.11	—	Razak *et al*.^35^
SSF	50 g/L OPEFB	3.97	0.16	0.03	Razali *et al*.^17^
DSSF	70 g/L sago hampas	4.62	0.11	0.06	Husin *et al*.^10^
Glucose	40 g/L glucose	5.33	0.13	—	Ibrahim *et al*.^19^
SHF	20 g/L OPEFB hydrolysate	1.69	0.08	—	Ibrahim *et al*.^19^
SSF	74 g/L Kraft paper mill sludge	10.2	0.13	—	Guan *et al*.^36^
SSF	50 g/L alkali-pretreated switchgrass	7.8	0.16	—	Guan *et al*.^31^

## Conclusions

The saccharification of fibrous lignocellulosic biomass such as OPEFB by cellulase in bioreactor should be conducted using pitched turbine impeller without the baffle, which produced 31 g/L of sugar. The SSF operated in a 2-L bioreactor produced 2.88 g/L of biobutanol similar to the SHF that produced 2.86 g/L. Introducing the DYEF in the SSF had improved biobutanol production by 46% to a concentration of 4.41 g/L. The *in-situ* recovery integrated into SSF with DYEF had improved the biobutanol production to 4.96 g/L. In overall, the SSF with DYEF and *in-situ* recovery had improved the normal SSF by 72%.

## Supplementary information

Supplementary document

## References

[1] Ibrahim MF, Ramli N, Kamal Bahrin E, Abd-Aziz S (2017). Cellulosic biobutanol by Clostridia: Challenges and improvements. Renew. Sustain. Energy Rev..

[2] Green EM (2011). Fermentative production of butanol—the industrial perspective. Curr. Opin. Biotechnol..

[3] Lee SY (2008). Fermentative butanol production by clostridia. Biotechnol. Bioeng..

[4] Jin C, Yao M, Liu H, Lee CF, Ji J (2011). Progress in the production and application of n-butanol as a biofuel. Renew. Sustain. Energy Rev..

[5] Zheng J, Tashiro Y, Wang Q, Sonomoto K (2015). Recent advances to improve fermentative butanol production: genetic engineering and fermentation technology. J. Biosci. Bioeng..

[6] Comyns AE (2010). Biobutanol: a future fuel?. Focus Catal..

[7] Noorshamsiana AW, Nur Eliyanti AO, Fatiha I, Astimar AA (2017). A review on extraction processes of lignocellulosic chemicals from oil palm biomass. J. Oil Palm Res..

[8] Zainal NH, Jalani NF, Mamat R, Astimar AA (2017). A review on the development of palm oil mill effluent (POME) final discharge polishing treatments. J. Oil Palm Res..

[9] Ibrahim MF, Abd-Aziz S, Razak MNA, Phang LY, Hassan MA (2012). Oil palm empty fruit bunch as alternative substrate for acetone-butanol-ethanol production by Clostridium butyricum EB6. Appl. Biochem. Biotechnol..

[10] Husin, H., Ibrahim, M. F., Kamal Bahrin, E. & Abd-Aziz, S. Simultaneous saccharification and fermentation of sago hampas into biobutanol by Clostridium acetobutylicum ATCC 824. *Energy Sci*. *Eng*. 1–11 (2018).

[11] García V, Päkkilä J, Ojamo H, Muurinen E, Keiski RL (2011). Challenges in biobutanol production: How to improve the efficiency?. Renew. Sustain. Energy Rev..

[12] Balan V (2014). Current Challenges in Commercially Producing Biofuels from Lignocellulosic Biomass. ISRN Biotechnol..

[13] Ibrahim MF, Abd-Aziz S, Yusoff MEM, Phang LY, Hassan MA (2015). Simultaneous enzymatic saccharification and ABE fermentation using pretreated oil palm empty fruit bunch as substrate to produce butanol and hydrogen as biofuel. *Renew*. Energy.

[14] Shah MM, Lee YY (1994). Process improvement in acetone-butanol production from hardwood by simultaneous saccharification and extractive fermentation. Appl. Biochem. Biotechnol..

[15] Li X, Li Z, Zheng J, Shi Z, Li L (2012). Yeast extract promotes phase shift of bio-butanol fermentation by Clostridium acetobutylicum ATCC824 using cassava as substrate. Bioresour. Technol..

[16] Umikalsom MS, Ariff AB, Karim MIA (1998). Saccharification of pretreated oil palm empty fruit bunch fiber using cellulase of Chaetomium globosum. J. Agric. Food Chem..

[17] Md Razali N (2018). Optimisation of Simultaneous Saccharification and Fermentation (SSF) for Biobutanol Production Using Pretreated Oil Palm Empty Fruit Bunch. Molecules.

[18] Xue C (2013). Two-stage *in situ* gas stripping for enhanced butanol fermentation and energy-saving product recovery. Bioresour. Technol..

[19] Ibrahim MF (2015). Effect of Buffering System on Acetone-Butanol-Ethanol Fermentation by Clostridium acetobutylicum ATCC 824 using Pretreated Oil Palm Empty Fruit Bunch. BioResources.

[20] Stanbury, P. F., Whitaker, A. & Hall, S. J. *Principle of fermentation technology*. (Butterworth-Heinemann, 1995).

[21] Shah MM, Song SK, Lee YY, Torget R (1991). Effect of pretreatment on simultaneous saccharification and fermentation of hardwood into acetone/butanol. Appl. Biochem. Biotechnol..

[22] Maddox IS, Steiner E, Hirsch S, Wessner S (2000). The cause of “ acid crash” and “ acidogenic fermentations” during the batch acetone-butanol-ethanol (ABE) fermentation process. J. Mol. Microbiol. Biotechnol..

[23] Kristensen JB, Felby C, Jørgensen H (2009). Yield-determining factors in high-solids enzymatic hydrolysis of lignocellulose. Biotechnol. Biofuels.

[24] Qureshi N (2010). Production of butanol (a biofuel) from agricultural residues: Part II – Use of corn stover and switchgrass hydrolysates✩. Biomass and Bioenergy.

[25] Sun Z, Liu S (2012). Production of n-butanol from concentrated sugar maple hemicellulosic hydrolysate by Clostridia acetobutylicum ATCC824. Biomass and Bioenergy.

[26] Wiehn, M., Staggs, K., Wang, Y. & Nielsen, D. R. *In situ* butanol recovery from Clostridium acetobutylicum fermentations by expanded bed adsorption. *Biotechnol*. *Prog*. **30**, 68–78 (2013). 10.1002/btpr.1841 24504855

[27] Xue C (2016). Butanol production in acetone-butanol-ethanol fermentation with *in situ* product recovery by adsorption. Bioresour. Technol..

[28] Mariano AP, Qureshi N, Maciel Filho R, Ezeji TC (2012). Assessment of *in situ* butanol recovery by vacuum during acetone butanol ethanol (ABE) fermentation. J. Chem. Technol. Biotechnol..

[29] Roffler SR, Blanch HW, Wilke CR (1987). *In-situ* recovery of butanol during fermentation. Bioprocess Eng..

[30] Ibrahim MF, Kim SW, Abd-Aziz S (2018). Advanced bioprocessing strategies for biobutanol production from biomass. Renew. Sustain. Energy Rev..

[31] Guan Wenjian, Shi Suan, Blersch David (2018). Effects of Tween 80 on fermentative butanol production from alkali-pretreated switchgrass. Biochemical Engineering Journal.

[32] Rizal NFAA (2018). Combination of Superheated Steam with Laccase Pretreatment Together with Size Reduction to Enhance Enzymatic Hydrolysis of Oil Palm Biomass. Molecules.

[33] Linggang S, Phang LY, Wasoh H, Abd-Aziz S (2013). Acetone–butanol–ethanol production by Clostridium acetobutylicum ATCC 824 using sago pith residues hydrolysate. BioEnergy Res..

[34] Shao, M. & Chen, H. Feasibility of acetone-butanol-ethanol (ABE) fermentation from Amorphophallus konjac waste by Clostridium acetobutylicum ATCC 824. *Process Biochem*. **50**, 1301–1307 (2015).

[35] Razak MNA, Ibrahim MF, Yee PL, Hassan MA, Abd-Aziz S (2013). Statistical optimization of biobutanol production from oil palm decanter cake hydrolysate by clostridium acetobutylicum ATCC 824. BioResources.

[36] Guan W, Shi S, Tu M, Lee YY (2016). Acetone-butanol-ethanol production from Kraft paper mill sludge by simultaneous saccharification and fermentation. Bioresour. Technol..

