# Supplementary material for: Improved Biobutanol Production in 2-L Simultaneous Saccharification and Fermentation with Delayed Yeast Extract Feeding and in-situ Recovery
Source: Sci Rep. 2019 May 15;9:7443. doi: 10.1038/s41598-019-43718-1 (PMC6520356; doi:10.1038/s41598-019-43718-1)
Supplement: Supplementary file 1 — Supplementary document [file 41598_2019_43718_MOESM1_ESM.pdf]

Improved Biobutanol Production in 2-L Simultaneous Saccharification and Fermentation with Delayed Yeast Extract Feeding and *in-situ* Recovery

Muhammad Siddiq Mohamed Salleh[1], Mohamad Faizal Ibrahim*[1,2], Ahmad Muhaimin Roslan[1,2], and Suraini Abd-Aziz[1]

[1]*Department of Bioprocess Technology, Faculty of Biotechnology and Biomolecular Sciences, Universiti Putra Malaysia, 43400 UPM Serdang, Selangor, Malaysia.*

[2]*Laboratory of Biopolymer and Derivatives, Institute of Tropical and Forestry and Forest Product, 43400 UPM Serdang, Selangor, Malaysia.*

siddiqsalleh@gmail.com; ar_muhaimin@upm.edu.my; suraini@upm.edu.my

*Corresponding author: faizal_ibrahim@upm.edu.my

Supplementary documents

S1: The observation during saccharification of oil palm empty fruit bunch in different types of impeller

**Type of impeller**	**Sugar production (g/L)**	**Observation**	**Observation remark**
Rushton turbine	21.00 ± 0.14	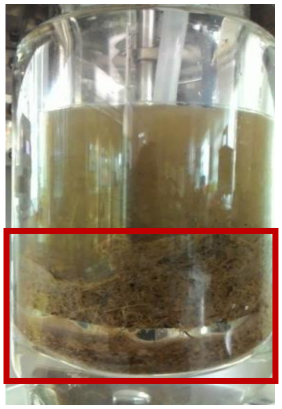	The OPEFB was not homogeneously mixed as the OPEFB starts to accumulate at the bottom of the bioreactor
Pitched turbine	27.22 ± 0.42	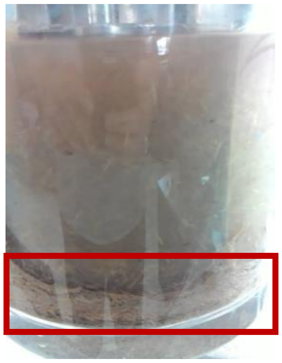	There is some amount of OPEFB trapped at the baffle, at the bottom of the bioreactor
Pitched turbine with lifted baffle ring to the middle of bioreactor	32.91 ± 0.53	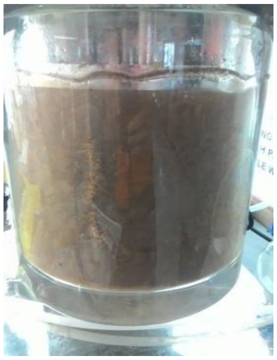	All OPEFB fibers were homogeneously mixed in the bioreactor
